# Trajectories of post-traumatic stress in sepsis survivors two years after ICU discharge: a secondary analysis of a randomized controlled trial

**DOI:** 10.1186/s13054-024-04815-4

**Published:** 2024-01-29

**Authors:** Konrad F. R. Schmidt, Jochen S. Gensichen, Maya Schroevers, Martina Kaufmann, Friederike Mueller, Gustav Schelling, Sabine Gehrke-Beck, Monique Boede, Christoph Heintze, Michel Wensing, Daniel Schwarzkopf

**Affiliations:** 1 Institute of General Practice and Family Medicine, Charité University Medicine, Charitéplatz 1, D-10117 Berlin, Germany; 2 Institute of General Practice and Family Medicine, Jena University Hospital, D-07743 Jena, Germany; 3 Center of Sepsis Control and Care (CSCC), Jena University Hospital, D-07747 Jena, Germany; 4 Institute of General Practice and Family Medicine, University Hospital of the Ludwig-Maximilians-University Munich, D-80336 Munich, Germany; 5 Department of Health Sciences, University Medical Center Groningen, University of Groningen, NL-9700 AB Groningen, The Netherlands; 6 Office of Good Scientific Practice, Charité University Medicine, D-10117 Berlin, Germany; 7 Department of Child and Youth Psychiatry, Psychosomatics and Psychotherapy, Asklepios Hospital Luebben, D-15907 Luebben, Germany; 8 Department of Anesthesiology, University Hospital of the Ludwig-Maximilians-University Munich, D-80336 Munich, Germany; 9 Department of General Practice and Health Services Research, University Hospital Heidelberg, D-69120 Heidelberg, Germany; 10 Department of Anesthesiology and Intensive Care Medicine, Jena University Hospital, Am Klinikum 1, D-07747 Jena, Germany

## Abstract

**Background:**

Post-traumatic stress has been identified as a frequent long-term complication in survivors of critical illnesses after sepsis. Little is known about long-term trajectories of post-traumatic stress and potentially modifiable risk factors following the ICU stay. Study objective was to explore and compare different clinical trajectories of post-traumatic stress symptoms in sepsis survivors up to two years after discharge from ICU.

**Methods:**

Data on post-traumatic stress symptoms by means of the Post-traumatic Symptom Scale (PTSS-10) were collected in sepsis survivors at one, six, 12 and 24 months after discharge from ICU. Data on chronic psychiatric diagnoses prior ICU were derived from the primary care provider’s health records, and data on intensive care treatment from ICU documentation. Trajectories of post-traumatic symptoms were identified ex post, discriminating patterns of change and k-means clustering. Assignment to the trajectories was predicted in multinomial log-linear models.

**Results:**

At 24 months, all follow-up measurements of the PTSS-10 were completed in *N* = 175 patients. Three clusters could be identified regarding clinical trajectories of PTSS levels: stable low symptoms (*N* = 104 patients [59%]), increasing symptoms (*N* = 45 patients [26%]), and recovering from symptoms (*N* = 26 patients [15%]). Patients with initially high post-traumatic symptoms were more likely to show a decrease (OR with 95% CI: 1.1 [1.05, 1.16]). Females (OR = 2.45 [1.11, 5.41]) and patients reporting early traumatic memories of the ICU (OR = 4.04 [1.63, 10]) were at higher risk for increasing PTSS levels.

**Conclusion:**

Post-traumatic stress is a relevant long-term burden for sepsis patients after ICU stay. Identification of three different trajectories within two years after ICU discharge highlights the importance of long-term observation, as a quarter of patients reports few symptoms at discharge yet an increase in symptoms in the two years following. Regular screening of ICU survivors on post-traumatic stress should be considered even in patients with few symptoms and in particular in females and patients reporting traumatic memories of the ICU.

**Supplementary Information:**

The online version contains supplementary material available at 10.1186/s13054-024-04815-4.

## Introduction

With advances in intensive care, the survival rate of critical illnesses as sepsis has increased [1, 2]. As a result, there is growing concern about the long-term impact on health-related quality of life after discharge from the intensive care unit (ICU) [3]. Survivors of critical illness often suffer from cognitive, mental and physical impairments, summarized as the Post-Intensive Care Syndrome (PICS) [4]. Within this, a large body of literature has found that more than one in five critical illness survivors may show clinical symptoms of depression and/ or Post-traumatic Stress Disorder (PTSD) [5–7]. Health-related quality of life (HRQOL) may be reduced for months and years [3, 8]. Therefore, the “International Guidelines for Management of Sepsis” [9] recommend continuous follow-up. However, there is still a lack of specific aftercare programs [10], while existing post-ICU clinics were not clearly found to be effective [11]. Additionally, mental health care often is hampered by structural capacity deficits in the provision of psychotherapy [12].

A recent meta-analysis [13] showed that mental trauma in medical populations is significantly more relevant than previously thought. In particular, sepsis survivors, who are affected by invasive medical care, show high rates of clinically significant PTSD symptoms [14, 15]. However, most existing studies are limited by small sample sizes [16–18], cross-sectional design [16] or short duration of follow-up [18]. Furthermore, only few of these studies specifically examined potential risk factors for post-sepsis PTSD [14, 16, 19].

In light of the growing evidence of adverse health and functional outcomes associated with post-traumatic symptoms [20, 21], and the high annual incidence of sepsis [2] describing the trajectories and potentially modifiable risk factors for adverse mental health outcomes after sepsis has important implications for population health, for example to identify patient groups at particular risk [22].

Aim of this study was to identify and predict trajectories of post-traumatic stress symptoms over two years in a cohort of sepsis survivors.

## Methods

### Study design and context

A retrospective observational cohort study on post-traumatic stress symptoms after sepsis was performed as a secondary analysis. Data were gathered as part of the SMOOTH-study (*Sepsis survivors MOnitoring and coordination in Outpatient healTH care)* [23], a multicenter, non-blinded, two-armed randomized clinical trial. Core components of the SMOOTH-trial’s intervention included post-ICU-discharge case management focusing on proactive patient symptom monitoring, clinical decision support for the primary care physicians by a consulting physician and training for both patients and their primary care physicians in evidence-based post-sepsis care. The trial was approved by the institutional review board of the Jena University Hospital (No.3001/111). Detailed methods and results of the SMOOTH-trial are described elsewhere [12, 23–25]. This secondary analysis identified different clusters ex post in the trajectories of post-traumatic stress symptoms among the SMOOTH patients. Predictors of these trajectory clusters were assessed by regression analysis.

### Sample

Patients were recruited in nine ICU study centers across Germany between February 2011 and December 2013. Follow-up assessments were completed in December 2015. Patients were eligible for inclusion if they were adult (≥ 18 years) survivors of severe sepsis or septic shock, (now defined as “sepsis”) [26] and fluent in the German language. Clinical diagnoses of sepsis were made by intensivists according to *American College of Chest Physicians/ Society of Critical Care Medicine* consensus criteria [27]. The key exclusion criterion was cognitive impairment that would prevent participation in the intervention, being assessed by the modified *Telephone Interview of Cognitive Status* (TICS-M; score ≤ 27) within one month after discharge [28].

### Procedure

Baseline data were collected through standardized face-to-face interviews with patients within one month of ICU discharge. Further clinical data were obtained from their ICU records. Follow-up data were collected by standardized telephone interviews at 6, 12 and 24 months after enrolment. All interviews were conducted by trained study nurses using a standardized interview guide.

### Measures

The primary outcome was post-traumatic stress which was assessed by telephone interviews using the *Post-traumatic Symptom Scale* (PTSS-10). This questionnaire assesses 10 major post-traumatic symptoms on a 7-point Likert scale, such as nightmares, irritability or fear of places and situations. It has been shown to be a responsive, valid and reliable instrument in screening for post-traumatic symptoms. The total sum score of the 10 items shows good internal consistency and test–retest reliability (range, 10–70; higher scores indicate greater impairment, scores above 35 are considered to indicate a PTSD diagnosis, scores above 23 to be clinically relevant) [29, 30]. However, the PTSS-10 does not replace a clinical interview to make a clinically confirmed diagnosis of a PTSD.

Based on the four assessments of the PTSS-10, different groups of longitudinal trajectories of post-traumatic symptoms were identified, see statistical analysis. To predict these trajectory groups, a set of 15 possible predictor variables was derived from literature, clinical reasoning, and availability of measures collected during the SMOOTH-trial. Only measures collected one month after ICU discharge were considered, to achieve a temporal sequence of predictors and trajectories. Predictors considered were:

- Patient demographics: age, sex, education, marital status [21, 31–34].
- Pre-existing comorbidities: presence of a diagnosis from chapter F of ICD-10 (mental and behavioral disorders) [32, 35–37], *Charlson Comorbidity Index* (range of possible scores, 0–37; high score indicates high impairment) [38].
- Extend of intensive care and severity of critical illness: mechanical ventilation, renal replacement therapy, ICU length-of-stay, number of ICD-10 diagnoses at discharge [6, 33, 39, 40].
- Symptoms at one-month follow-up: pain intensity as assessed by the *Graded Chronic Pain Scale* (GCPS, range of possible scores, 0–100; high score indicates high impairment) [41, 42], cognitive function [43] assessed by the TICS-M (range of possible scores, 0–50; includes only scores above 27 by inclusion criterion; high score indicates low impairment) [28] presence of at least 2 of 4 types of traumatic memories of the ICU experience as measured by the corresponding additional items of the PTSS-10 [44]
- Randomization status (control vs. intervention) was added as a control variable, as the intervention had an effect on the PTSS level at the 24-month follow-up [25].
- The first measurement of the PTSS-10 at the one-month follow-up was included as a control variable, as trajectories over time are influenced by their initial levels (ceiling and floor effects, regression to the mean).

### Statistical analysis

All analyses were performed using R version 4.1.2 statistical software [45]. Each patient's four follow-up PTSS-10 sum scores defined the patient's individual longitudinal trajectory of post-traumatic stress symptoms. We conducted an exploratory analysis to identify different clusters of individual trajectories. In order to do this, we followed the three-step procedure proposed by Leffondre et al. [46, 47] which makes no assumptions about specific trajectory shapes. This procedure involves (1) calculating 24 measures describing the characteristics of the trajectories; (2) using factor analysis to select the most important subset of the 24 measures and (3) using cluster analysis based on these measures to identify clusters of trajectories, and classify each individual trajectory into one of the clusters. Steps 1 and 2 were performed using the *traj* package (functions *step1measures* and *step2factors*). The number of clusters (step 3) was determined using the *NbClust* package (function *NbClust* with k-means clustering), which provides 26 fit indices for the number of clusters. The number of clusters, for which the best fit was indicated by a relative majority of the indices, was selected. Finally, the *step3clusters* function from the *traj* package was used to assign each individual trajectory to one of the clusters by k-means clustering. To describe the clusters, the individual trajectories as well as the median and interquartile range of the PTSS score per cluster were visualized for the whole sample as well as stratified by intervention and control group, see Additional file 3.

Identified clusters were compared descriptively with respect to the predictor variables given above. The significance of the predictor and control variables was assessed in two steps: (1) calculation of a multinomial log-linear model for each predictor, controlling for the PTSS-10 sum score measured at the one-month follow-up; (2) inclusion of all predictors significant at *P* ≤ 0.05 in step 1 in a multiple multinomial log-linear model, also controlling for the effect of the PTSS-10 sum score at the one-month follow-up. Multinomial log-linear models were calculated using the *multinom* function of the *nnet* package.

As the trajectory clustering analysis required at least four measurements for each trajectory, only those patients were included for whom data on post-traumatic stress were available for all four measurements. Analyses were performed on complete data. Patterns of missing data were analyzed descriptively to assess possible effects of missing data, see Additional File 2: Fig. 2. All statistical tests were performed at a two-sided alpha level of 0.05.

## Results

### Longitudinal trajectories of post-traumatic stress symptoms

At 24 months after discharge from ICU, *N* = 186 (64%) of initially *N* = 291 enrolled patients completed the 24-month follow-up, *N *= 64 patients have died and *N* = 41 patients were excluded or dropped out for other reasons. *N* = 175 of these *N* = 186 patients (94%) provided all four follow-up PTSS-10 assessments and were included in the analysis, see Additional file 1: Fig. 1.

Of the 175 included patients, 94 were in the intervention group and 81 in the control group. The 116 excluded patients were older and had comorbidity with lower cognitive function, see Additional File 4: Table S1). The pattern of missing values for PTSS-10 was mostly monotonic due to loss to follow-up, see Additional File 2: Fig. 2.

**Fig. 1:**
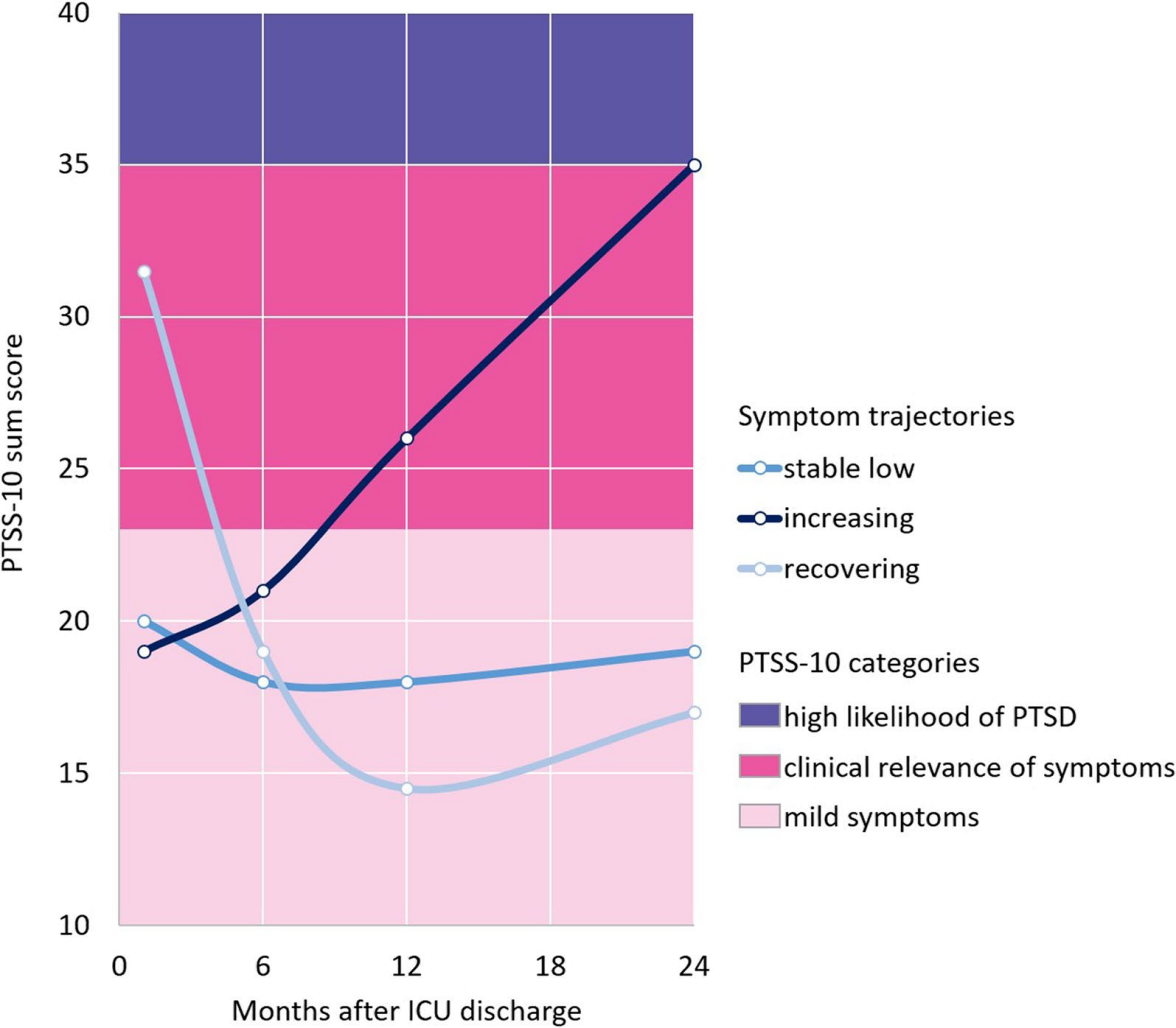
Three main trajectories of post-traumatic symptoms up to 24 months after ICU care were identified in sepsis survivors (means). The recovering cluster, on average, started with clinically relevant symptoms that decreased clearly over time. In contrast, the increasing cluster started with mild symptoms and had a high likelihood of manifest PTSD at 24 months

**Fig. 2:**
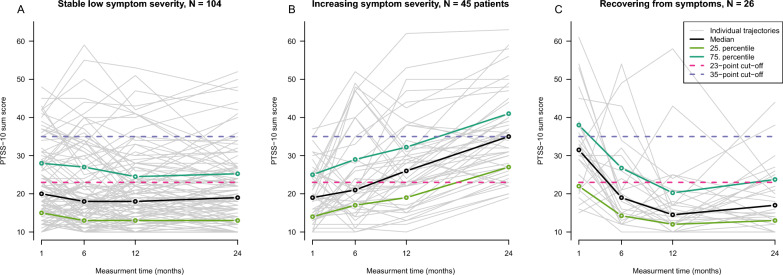
Clusters of trajectories of post-traumatic symptoms. Panel A: cluster is interpreted as stable low severity of symptoms, *N* = 104 patients. Panel B: cluster is interpreted as increasing severity of symptoms, *N *= 45 patients. Panel C: cluster is interpreted as recovering from symptoms, *N* = 26 patients. Sum scores above 35 are considered to indicate PTSD, above 23 to be clinically relevant

Out of 26 fit indices, a relative majority of 12 indices suggested the number of three trajectory clusters as optimal solution, see Fig. 1. The three trajectories can be interpreted as stable low (panel A, *N* = 104 patients [59%]), increasing (panel B, *N* = 45 patients [26%]), and recovering to normal level (panel C, N = 26 patients [15%]) with respect to the development of post-traumatic symptoms, see Fig. 2. The recovering cluster had a higher initial PTSS-10 sum score than the stable low and increasing clusters (median 31.5 vs. 20 and 19, respectively). The increasing cluster showed a median PTSS-10 sum score of 35 at 24 months, which indicate a high likelihood of PTSD. The three trajectory clusters were comparable between the intervention and control groups, see Additional file 3.

### Prediction of trajectories

Table 1 shows the descriptive comparison of predictor variables between the three clusters without adjustment for the initial severity of post-traumatic symptoms. Table 2 provides results of the multinomial log-linear models: Controlling for the effect of the initial symptom severity (i.e., PTSS-10 at one month), randomization to the intervention group, female gender and the presence of more than two traumatic ICU memories at one-month follow-up, showed significant individual effects (*p* ≤ 0.036). These effects were confirmed after inclusion in the multivariate model, see Table 3: Patients in the intervention group and those with higher initial post-traumatic symptoms were more likely to be in the recovering group (OR with 95% CI = 1.44 [0.54, 3.86] and 1.1 [1.05, 1.16], respectively) and less likely to be in the increasing group (OR = 0.33 [0.15, 0.75], and 0.93 [0.89, 0.98], respectively). In contrast, female patients (OR = 2.45 [1.11, 5.41]) and patients reporting more than two traumatic memories (OR = 4.04 [1.63, 10]) were more likely to show increasing than stable low symptoms.

**Table 1:** Comparison of patient characteristics between the clusters of individual post-traumatic stress trajectories

Predictor variables	Included into analysis (*N* = 175)	Clusters of individual post-traumatic stress trajectories (*N* = 175)			
	(No. of patients with complete data)	Stable low *N* = 104 (59.4%)	Increasing *N* = 45 (25.7%)	Recovering *N* = 26 (14.9%)	*P*-value
Intervention group	94 (53.7%) (N = 175)	60 (57.7%)	17 (37.8%)	17 (65.4%)	**0.035**
Age (years)	61 [51.5, 72] (N = 175)	62 [51.75, 1.25]	58 [51, 72]	60.5 [53, 71.75]	0.793
Female sex	57 (32.6%) (N = 175)	28 (26.9%)	23 (51.1%)	6 (23.1%)	**0.008**
Higher education	44 (25.1%) (N = 175)	24 (23.1%)	10 (22.2%)	10 (38.5%)	0.236
Married	92 (53.2%) (N = 173)	52 (50.5%)	27 (60%)	13 (52%)	0.561
ICD-10 F-diagnosis before ICU stay	22 (16.7%) (N = 132)	10 (12.7%)	9 (24.3%)	3 (18.8%)	0.283
*Charlson Comorbidity Index*	3 [1, 5] (N = 174)	3 [2, 5]	2 [1, 4]	3.5 [1, 6]	0.1
Mech. ventilation during ICU stay	148 (85.1%) (N = 174)	83 (80.6%)	40 (88.9%)	25 (96.2%)	0.097
Renal replacement therapy during ICU stay	48 (27.6%) (N = 174)	25 (24.3%)	14 (31.1%)	9 (34.6%)	0.475
ICU length-of-stay (days)	24 [13, 42.75] (N = 162)	23.5 [12, 42.8]	25.5 [16.3, 44]	19.5 [13, 35.5]	0.539
No. of ICD-10 diagnoses at discharge	9 [6, 14] (N = 166)	9 [6, 13]	9 [6, 16]	10 [6.5, 13.5]	0.525
Pain intensity at one month (GCPS)	50 [26.67, 60] (N = 173)	50 [27.5, 62.5]	43.33 [26.7, 53.3]	50 [30.8, 62.5]	0.513
Cognitive functioning at one month (TICS-M)	34 [31, 36] (N = 175)	34 [31, 36]	34 [32, 36]	34.5 [31, 36]	0.62
No. of traumatic memories of the ICU > 2 at one month	61 (35.1%) (N = 174)	29 (27.9%)	21 (47.7%)	11 (42.3%)	**0.048**
Severity of post-traumatic symptoms at one month(PTSS-10 sum score)	21 [15, 29.5] (N = 175)	20 [15, 28]	19 [14, 25]	31.5 [22, 38]	**≤ 0.001**

Descriptive statistics presented as median [1st quartile, 3rd quartile] or N (%). ICU: intensive care unit. GCPS: Graded Chronic Pain Scale. TICS-M: modified Telephone Interview for Cognitive Status. PTSS-10: Post-traumatic Symptom Scale. P-values obtained by Kruskal–Wallis test or χ^2^ test, as appropriate. *P*-values set in boldface indicate statistical significance

**Table 2:** Association of individual characteristics with cluster assignment

	Effects on risk of belonging to a cluster (odds ratios with 95% confidence interval)			Significance of predictor (*P*-value)
	Stable low (reference)	Increasing (vs. reference)	Recovering (vs. reference)	
Intervention group	1	0.44 (0.21, 0.9)	1.44 (0.55, 3.77)	**0.036**
Age	1	0.99 (0.97, 1.02)	1.02 (0.98, 1.06)	0.353
Female sex	1	3.02 (1.44, 6.35)	0.67 (0.23, 1.99)	**0.005**
Higher education	1	0.91 (0.39, 2.12)	3.04 (1.09, 8.53)	0.088
Married	1	1.47 (0.72, 3.01)	0.87 (0.33, 2.26)	0.503
ICD-10 F-diagnosis before ICU stay	1	2.62 (0.92, 7.52)	1.09 (0.24, 4.92)	0.196
*Charlson Comorbidity Index*	1	0.86 (0.74, 1)	0.94 (0.79, 1.11)	0.123
Mechanical ventilation during ICU stay	1	1.89 (0.66, 5.44)	5.39 (0.66, 43.78)	0.098
Renal replacement therapy during ICU stay	1	1.44 (0.66, 3.15)	1.64 (0.61, 4.44)	0.483
ICU length-of-stay (days)	1	1.01 (0.99, 1.02)	1.01 (0.99, 1.03)	0.618
Number of ICD-10 diagnoses at discharge	1	1.05 (0.98, 1.14)	1.02 (0.92, 1.13)	0.403
Pain intensity at one month (GCPS)	1	1 (0.98, 1.01)	0.98 (0.96, 1)	0.178
Cognitive functioning at one month (TICS-M)	1	1.04 (0.94, 1.15)	1.01 (0.9, 1.15)	0.772
Number of Traumatic memories of the ICU > 2 at one month	1	3.64 (1.59, 8.32)	0.77 (0.27, 2.24)	**0.004**

Results of individual multinomial log-linear models for each predictor variable. All models adjust for the PTSS-10 sum score measured at one month as a covariate. P-values obtained by likelihood ratio tests. ICU: intensive care unit. GCPS: Graded Chronic Pain Scale. TICS-M: modified Telephone Interview for Cognitive Status. PTSS-10: Post-traumatic Symptoms Scale. *P*-values set in boldface indicate statistical significance

**Table 3:** Multivariate analysis of predictors for cluster assignment

	Effects on risk of belonging to a cluster (odds ratios with 95% confidence interval)			Significance of predictor (*P*-value)
	Stable low (reference)	Increasing (vs. reference)	Recovering (vs. reference)	
Intervention group	1	0.33 (0.15, 0.75)	1.44 (0.54, 3.86)	**0.012**
Female sex	1	2.45 (1.11, 5.41)	0.69 (0.23, 2.05)	**0.048**
Number of Traumatic memories of the ICU > 2 at one month	1	4.04 (1.63, 10)	0.72 (0.24, 2.16)	**0.004**
Severity of post-traumatic symptoms at one month (PTSS-10 sum score)	1	0.93 (0.89, 0.98)	1.1 (1.05, 1.16)	** ≤ 0.001**

Results of multinomial log-linear model including all presented predictors. Model is based on *N* = 175 cases with complete data. Nagelkerke’s *R*^2^ of the model was 0.28. *P*-values obtained by likelihood ratio tests. ICU: intensive care unit. PTSS-10: Post-traumatic Symptoms Scale. *P*-values set in boldface indicate statistical significance

## Discussion

The aim of this secondary analysis of a randomized clinical trial was to explore trajectories of post-traumatic stress symptoms in sepsis survivors. Our sample was similar to other cohorts of sepsis survivors in terms of age, sex and comorbidities, but had longer duration of mechanical ventilation and ICU stay [24]. Excluded patients were older and had more comorbidities and lower cognitive function, which may be because most patients dropped out due to mortality.

We identified three clusters with a distinct trajectory of symptoms over two years of follow-up. The majority of patients showed rather stable and mostly low levels of post-traumatic symptoms. Other patients recovered from initially severe symptoms, and a third group showed a trajectory of increasing post-traumatic stress over time. In particular, female patients and patients who reported traumatic memories shortly after ICU discharge appeared to be at higher risk of increasing clinically relevant symptoms in the long-term.

Outside the ICU context, it has been widely shown, that post-traumatic symptoms and a clinically confirmed PTSD can follow different trajectories, which have been investigated in numerous studies following traumatic events such as war, military deployment, accidents, or violence. In a study of tsunami survivors (one, three and six years after the traumatic event), Johannesson et al. showed four long-term PTSD trajectories: resilient (72.3%), severe chronic (4.6%), moderate chronic (11.2%) and recovering (11.9%) [48]. In a meta-analysis of 54 studies, Galatzer-Levy et al. identified 12 different trajectory groups across studies, of which four were most commonly reported: chronic (12%, high symptoms over time), resilient (65%), delayed onset and recovering [49]. Across studies, they found a mean prevalence of 65% for resilient trajectories, which match the stable low group found in our sample (59%). In the meta-analysis, recovery had a mean prevalence of 23%, roughly consistent with our results (15%), and delayed onset occurred in 18%, consistent with our increasing pattern (26%). Our results are therefore broadly consistent with previous research on PTSD outside the ICU.

There is a paucity of research on the long-term course of PTSD in ICU survivors, as most previous studies included only one follow-up assessment [50]. Only two ICU related studies have described the longitudinal development of PTSD at group level [19, 51]. Both studies used predefined clinical criteria to define trajectory groups, whereas we—like most studies outside the ICU context—took an exploratory, data-driven approach to cluster trajectories [49, 51]: Bienvenu et al. followed ICU survivors for two years with four follow-up measurements: Based on the presence of clinically relevant symptoms they found four subgroups: no symptoms, maintainers, remitters and relapsers [51]. Most patients either reported no relevant or maintained clinically relevant PTSD symptoms over time, which corresponds to our stable low trajectory group. Remission was observed in approximately 14% of patients, which corresponds to the 15% of patients in our recovering trajectory. In contrast, the relapse group was small (4.8%) and showed little remission of symptoms over time. For a five-year follow-up, Bienvenu et al. only distinguished between maintaining/ recurring symptoms and no symptoms/ remitting symptoms [52] which makes comparisons with our data difficult. Wintermann et al. [19] measured PTSD symptoms at three and six months after discharge, corresponding roughly to the period between our first and second assessment. They found that delayed onset PTSD symptoms occurred in a quarter of patients—defined as the first occurrence of clinically relevant symptoms six months after discharge. This corresponds to our trajectory group of increasing symptoms. Our results also suggest that symptoms may continue to worsen in the second year after discharge. Taken together, these results may indicate that ICU treatment is associated with a higher risk of late-onset PTSD symptoms compared with other traumatic events.

Consistent with the meta-analysis by Parker et al. [6], which included follow-up data up to 12 months, we did not find that the duration or invasiveness of the ICU treatment predicted post-traumatic symptoms. The authors discussed that other factors, such as the type of sedation, may be more important in the development of traumatic memories in the ICU. Unlike other traumatic events, ICU patients usually do not experience a sudden, single trauma, rather than a cumulative traumatization due to the experienced helplessness, exposure to invasive medical interventions, severity of illness and medically induced sedation or altered states of consciousness, including delirium [53]. Continuity of experience may prevent emotional processing and limit the ability to integrate early traumatic memories. As a result, these memories are often implicit and fragmentary [54]. Similar to being awake under anesthesia, snippets of conversations, sounds, pain and other impressions are recalled which, cannot be placed in time and space and are later re-experienced as real [55]. In line with this, early memories of the ICU emerged as a significant predictor of increasing post-traumatic symptoms in our sample, possibly indicating the intensity of traumatization, consistent with literature [6, 30, 56]. In particular, also Wintermann et al. identified the number of traumatic memories as a predictor of late-onset PTSD [19]. Even if PTSD severity cannot be predicted by a single factor, early traumatic memories should be given more attention in screening procedures. This may be supported by the fact, that ICU diaries or documentation by ICU nurses have been shown to help ICU survivors integrate and emotionally process traumatic experiences [57].

Although women have been shown to be at increased risk of PTSD [58, 59], long-term PTSD trajectories were rarely studied from a gender perspective [60]. In contrast to our findings, van Zuiden et al. demonstrated that women were more likely to recover from PTSD symptoms one year after a serious injury, whereas men were more likely to show a delayed symptom onset [31]. Consistent with our findings, Lowe et al. [32] showed in their pooled analysis of six longitudinal studies in adult survivors of civilian injuries that women were at higher risk of both initial post-traumatic symptoms and late onset. The authors suggest that both the nature of the trauma and the intensity of the acute emotional response may account for the disproportionate risk of PTSD in women. Other authors discuss sex differences in brain neurocircuitry, anatomy and neurobiological processes involved in memory consolidation [61, 62]. As (1) the consolidation of traumatic experiences depends on stress hormone levels [56, 63, 64] and (2) sepsis patients often show high inflammatory and neuroendocrine stress responses [15] or alterations in the endocannabinoid system anyway [40], sex-specific neurobiological interactions may also be relevant for our sample [64].

Our findings support both the need for timely screening for early traumatic memories after discharge from the ICU and for regular monitoring of post-traumatic stress symptoms in the long-term. As our results show that women have higher rates of post-traumatic stress symptoms and are also reported to respond better to treatment [65], female patients may receive particular attention. Particularly, GPs need to be aware of this issue, as they provide the most continuity of care for patients [66].

This study has several strengths: Long-term trajectories of post-traumatic symptoms in ICU survivors are still poorly described, even less so with multiple follow-ups and in sepsis survivors. In addition, the identification of individual risk factors has been barely studied. Our secondary analysis includes symptom assessment at four time points over a total period of two years, which even allows the identification of undulating trajectories. In addition, a wide range of individual risk factors for post-traumatic symptoms after sepsis could be examined, such as age, gender, socioeconomic status, pre-existing physical and psychological morbidity and intensive care parameters.

This study has limitations: It was an exploratory secondary analysis of trial data. In addition, patients in the intervention group received additional care that affected the outcome studied. Thus, we used randomization status as a covariate in the identification of predictors of outcome groups. At a descriptive level, the same clusters were found in both the intervention and control groups, see Additional file 3.

Grouping of longitudinal trajectories is a complex exploratory analysis that is susceptible to method bias, with different methods leading to different trajectory groups [67]. Therefore, findings need to be replicated in larger prospective cohorts with more measurement points, which would allow comparison of the results of different grouping methods [67, 68].

Finally, the use of the PTSS-10 for screening [30] does not allow to define a clinical diagnosis of a Post-Traumatic Stress Disorder (PTSD), which requires a detailed diagnostic interview by a psychiatrist. Consequently, patients with high PTSS-10 scores need to be referred for further psychiatric assessment.

Although our data are almost a decade old, they still appear to reflect the state of the art when compared in terms of clinical characteristics or intensive care procedures with current studies, such as an RCT by our research group on PTSD in critical illness survivors [69, 70].

## Conclusion

Post-traumatic stress is a relevant long-term burden for sepsis patients after ICU stay. This analysis of predictive trajectories supports both the identification of patients at risk for PTSD after sepsis and the importance of their long-term observation. Women in particular may be at risk of increasing symptom severity, and the presence of traumatic ICU memories could be used as an early warning sign for the development of PTSD.

Regular screening of sepsis survivors for post-traumatic stress symptoms should be considered, even in patients with few initial symptoms and beyond 12 months, as future worsening is possible. Given the high continuity of care in general practice, screening for symptoms may be best implemented in this setting.

## Supplementary Information

**Additional file1**: Flow chart of the study population

**Additional file2**: **Measures of post-traumatic stress over time:** Patterns of missing values In total, there were 116 patients with missing values on at least one measurement of the Post-traumatic Symptom Scale (PTSS-10). Horizontal bars present the number of patients with missing measurement for each time point, vertical bars present the number of patients with a specific pattern of missing values across all time points. For example, the largest pattern includes N=59 patients with missing values at the six months follow-up and all subsequent follow-ups as marked by the connected points below. The vast majority of missing values are monotonic, meaning that after a missing follow-up measurement, all subsequent follow-up measurements are also missing.

**Additional file3**: **Comparison of cluster-results between control and intervention group.** Panels A, C, and E show stable low, increasing, and recovering clusters for the control group. Panels B, D, and F present same clusters for the intervention group. Sum scores above 35 are considered to indicate PTSD, above 23 to be clinically relevant.

**Additional file4**:** Table S1: Descriptive statistics comparing included cases with drop-out.**

## Data Availability

The datasets used and/or analyzed during the current study are available from the corresponding author on reasonable request.

## Abbreviations

CI: Confidence Interval
HRQOL: Health-Related Quality of Life
ICU: Intensive Care Unit
OR: Odds ratio
PTSD: Post-traumatic Stress Disorder
PTSS: Post-traumatic Symptom Scale
