# Supplementary material for: Trajectories of post-traumatic stress in sepsis survivors two years after ICU discharge: a secondary analysis of a randomized controlled trial
Source: Crit Care. 2024 Jan 29;28:35. doi: 10.1186/s13054-024-04815-4 (PMC10823628; doi:10.1186/s13054-024-04815-4)
Supplement: Supplementary file 1 — Additional file1: Flow chart of the study population [file 13054_2024_4815_MOESM1_ESM.docx]

682 approached

160 met exclusion criteria

123 died

38 could not be reached

**291 included at baseline**

291 included at baseline

105 withdrew

64 died

11 refused for health reasons

18 refused for other reasons

12 could not be reached

186 completed 24 months follow up

**175** provided all four follow-ups

measurements of the PTSS-10

**– included into analysis**

94 received usual care

81 received interventional care
