# Supplementary figures and images for: Trajectories of post-traumatic stress in sepsis survivors two years after ICU discharge: a secondary analysis of a randomized controlled trial
Source: Crit Care. 2024 Jan 29;28:35. doi: 10.1186/s13054-024-04815-4 (PMC10823628; doi:10.1186/s13054-024-04815-4)

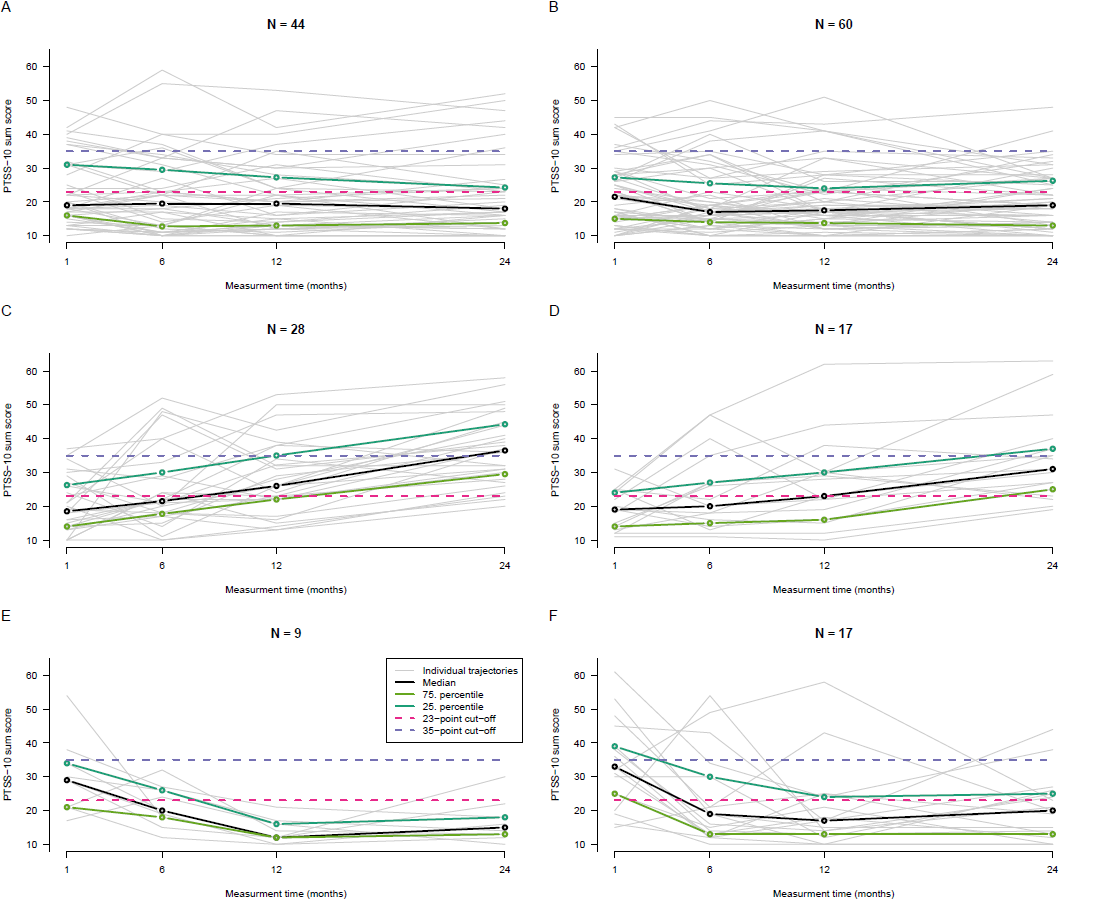

Supplement: Supplementary file 2 — Additional file2: Measures of post-traumatic stress over time: Patterns of missing values In total, there were 116 patients with missing values on at least one measurement of the Post-traumatic Symptom Scale (PTSS-10). Horizontal bars present the number of patients with missing measurement for each time point, vertical bars present the number of patients with a specific pattern of missing values across all time points. For example, the largest pattern includes N=59 patients with missing values at the six months follow-up and all subsequent follow-ups as marked by the connected points below. The vast majority of missing values are monotonic, meaning that after a missing follow-up measurement, all subsequent follow-up measurements are also missing. [file 13054_2024_4815_MOESM2_ESM.png]

Size of missingness pattern of PTSS-10 measurements over time (N)

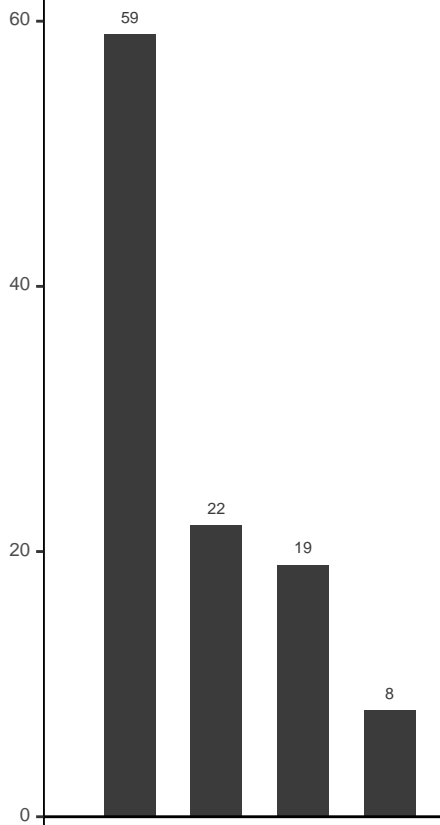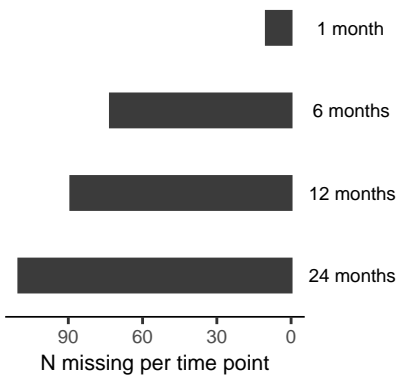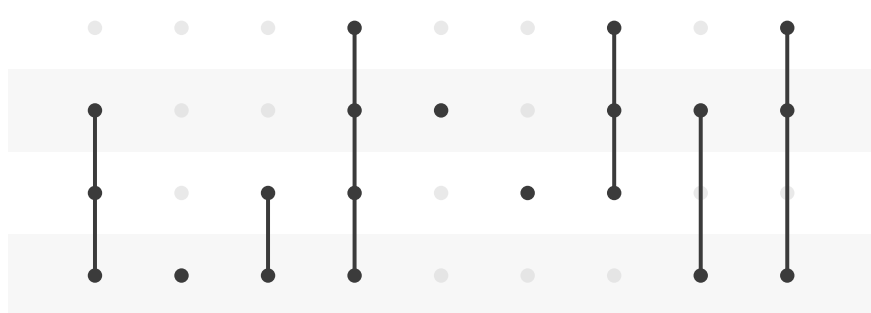

Supplement: Supplementary file 3 — Additional file3: Comparison of cluster-results between control and intervention group. Panels A, C, and E show stable low, increasing, and recovering clusters for the control group. Panels B, D, and F present same clusters for the intervention group. Sum scores above 35 are considered to indicate PTSD, above 23 to be clinically relevant. [file 13054_2024_4815_MOESM3_ESM.pdf]
