# Supplementary material for: Trajectories of post-traumatic stress in sepsis survivors two years after ICU discharge: a secondary analysis of a randomized controlled trial
Source: Crit Care. 2024 Jan 29;28:35. doi: 10.1186/s13054-024-04815-4 (PMC10823628; doi:10.1186/s13054-024-04815-4)
Supplement: Supplementary file 4 — Additional file4: Table S1: Descriptive statistics comparing included cases with drop-out. [file 13054_2024_4815_MOESM4_ESM.docx]

**Additional file 4: Table S1: Descriptive statistics comparing included cases with drop-outs**

| **Variables** | **N cases with available data** | **All cases,**  **N = 291** | **Drop-out,**  **N = 116** | **Included,**  **N = 175** | **P value** |
| --- | --- | --- | --- | --- | --- |
| Intervention group | 291 | 148 (50.9%) | 54 (46.6%) | 94 (53.7%) | 0.281 |
| Age (years) | 290 | 64 [53, 72] | 67 [56, 73] | 61 [51.5, 72] | **0.029** |
| Female sex | 290 | 96 (33.1%) | 39 (33.9%) | 57 (32.6%) | 0.899 |
| Higher education | 291 | 67 (23%) | 23 (19.8%) | 44 (25.1%) | 0.322 |
| Married | 284 | 148 (52.1%) | 56 (50.5%) | 92 (53.2%) | 0.715 |
| ICD-10 F-diagnosis before ICU stay | 208 | 42 (20.2%) | 20 (26.3%) | 22 (16.7%) | 0.108 |
| Charlson Comorbidity Index | 289 | 4 [2, 6] | 5 [2, 7] | 3 [1, 5] | **<=0.001** |
| Mech. ventilation during ICU stay | 289 | 244 (84.4%) | 96 (83.5%) | 148 (85.1%) | 0.742 |
| Renal replacement therapy during ICU stay | 288 | 82 (28.5%) | 34 (29.8%) | 48 (27.6%) | 0.691 |
| ICU length-of-stay (days) | 264 | 26 [13, 46] | 32 [15, 56.5] | 24  [13, 42.75] | **0.043** |
| No. of ICD-10 diagnoses at discharge | 278 | 9 [6, 14] | 10 [6, 14] | 9  [6, 14] | 0.442 |
| Pain intensity at one month (GCPS) | 276 | 46.67 [26.67, 60] | 46.67 [30, 60] | 50  [26.67, 60] | 0.654 |
| Cognitive functioning at one month (TICS-M) | 289 | 34 [31, 36] | 32 [30, 35] | 34 [31, 36] | **0.009** |
| No. of traumatic memories of the ICU >2 at one month | 278 | 103 (37.1%) | 42 (40.4%) | 61 (35.1%) | 0.441 |
| Severity of post-traumatic symptoms at one month  (PTSS-10 sum score) | 281 | 22  [15, 31] | 23  [16, 31.75] | 21  [15, 29.5] | 0.284 |

Descriptive statistics presented as median [1^st^ quartile, 3^rd^ quartile] or N (%). ICU: intensive care unit. GCPS: Graded Chronic Pain Scale. TICS-M: modified Telephone Interview for Cognitive Status. PTSS-10: Post-traumatic Symptom Scale.
